# Alternative methylation of intron motifs is associated with cancer-related gene expression in both canine mammary tumor and human breast cancer

**DOI:** 10.1186/s13148-020-00888-4

**Published:** 2020-07-21

**Authors:** A-Reum Nam, Kang-Hoon Lee, Hyeon-Ji Hwang, Johannes J. Schabort, Jae-Hoon An, Sung-Ho Won, Je-Yoel Cho

**Affiliations:** 1 Department of Biochemistry, BK21 Plus and Research Institute for Veterinary Science, School of Veterinary Medicine, Seoul National University, Gwanak-ro1, Gwanak-gu, Seoul, Korea; 2 Department of Public Health Sciences, Graduate School of Public Health, Seoul National University, Seoul, Korea

**Keywords:** Canine mammary gland tumor, Human breast cancer, Methylome, Transcriptome, Comparative study

## Abstract

**Background:**

Canine mammary tumor (CMT) has long been considered as a good animal model for human breast cancer (HBC) due to their pathological and biological similarities. However, only a few aspects of the epigenome have been explored in both HBC and CMT. Moreover, DNA methylation studies have mainly been limited to the promoter regions of genes.

**Results:**

Genome-wide methylation analysis was performed in CMT and adjacent normal tissues and focused on the intron regions as potential targets for epigenetic regulation. As expected, many tumor suppressors and oncogenes were identified. Of note, most cancer-associated biological processes were enriched in differentially methylated genes (DMGs) that included intron DMRs (differentially methylated regions). Interestingly, two PAX motifs, PAX5 (tumor suppressive) and PAX6 (oncogenic), were frequently found in hyper- and hypomethylated intron DMRs, respectively. Hypermethylation at the PAX5 motifs in the intron regions of *CDH5* and *LRIG1* genes were found to be anti-correlated with gene expression, while *CDH2* and *ADAM19* genes harboring hypomethylated PAX6 motifs in their intron region were upregulated. These results were validated from the specimens originally MBD-sequenced as well as additional clinical samples. We also comparatively investigated the intron methylation and downstream gene expression of these genes using human breast invasive carcinoma (BRCA) datasets in TCGA (The Cancer Genome Atlas) public database. Regional alteration of methylation was conserved in the corresponding intron regions and, consequently, gene expression was also altered in HBC.

**Conclusions:**

This study provides good evidence for the conservation of epigenetic regulation in CMT and HBC, and suggests that intronic methylation can be an important factor in better understanding gene regulation in both CMT and HBC.

## Introduction

Breast cancer (BC) is the most frequently diagnosed and the second leading cause of cancer death in woman worldwide [1]. The comparison of 5-year survival rates between cancer stages 4 and 2, 27% vs. 99% in the USA, clearly shows that earlier diagnosis is crucial for increasing patient survival [2]. Many BC risk factors have been reported; some are uncontrollable, such as old age and gene mutations, while some are controllable, such as diet and smoking [3]. Only about 5–10% of BCs are thought to be hereditary [4]. Representatively, inherited mutations in *BRCA1 and BRCA2*, which have roles in DNA repair, have been known as the most common cause of hereditary BC [5]. In addition to inherited mutations, somatic mutations of dozens of genes, including *CCND1*, *ERBB2*, *PIK3CA*, and *PTEN*, have been revealed as driver mutations that can lead to functional abnormalities and initiate breast tumorigenesis [6, 7]. The fast-growing databases of various human cancers, such as COSMIC and TCGA, now provide researchers with access to genomic data to test their hypothesis in clinical samples (https://cancer.sanger.ac.uk/cosmic; https://www.cancer.gov/tcga) [8, 9]. On the other hand, the molecular biological effects of environmental factors such as smoking, diet, and exercise [3] are not readily accessible in BC and further approaches are needed to investigate epigenomic changes, including DNA methylation [10].

The association of CpG dinucleotide DNA methylation with cancer-related phenotypes [11] is well understood in various types and at all stages of cancer progression [12, 13]. Hypermethylation, which has been known to be associated with repressed gene expression of tumor suppressors, is one of the important paradigms of carcinogenesis [14] and is supported by the activated mutations of DNA methyltransferases (DNMTs) being oncogenic in several tissues [15]. In various human cancers, genome-wide methylation has been profiled [14] and global DNA hypomethylation [16], along with local hyper- (tumor suppressors) and hypo- (oncogenes) methylations concomitant with the respective silencing and activating of gene expression [17, 18] were reported and suggested as potential diagnostic and predictive biomarkers [19]. The use of methylation alteration as a biomarker has several obvious advantages, such as early detection and relative specimen stability, but only a few are currently clinically used (e.g., methylation of *MGMT* in glioblastoma, *SEPT9* in hepatocellular carcinoma, and *PITX2* in breast cancer) [20].

Very similar to BC in human, canine mammary tumor (CMT) is one of the most common cancers in female dogs [21]. Clinical and pathophysiological similarities existing between HBC and CMTs are well-documented, including the spontaneous tumor incidence, comparable onset age, hormonal etiology, and the identical course of the disease [21]. Furthermore, CMT’s molecular characteristics, including several subtype molecular markers such as steroid receptor, epidermal growth factor (EGF), and proliferation markers, are also similar to HBC [22]. Recently, we reported a transcriptome signature in CMT [23] and other high-throughput sequencing studies on the aspects of CMT have been reported [24, 25]. However, no comprehensive genome-wide methylome profiles that are comparable to studies in HBC have been uncovered yet.

In the present study, we profiled the CMT-associated genome-wide methylation signature using methyl CpG binding domain (MBD) sequencing. In particular, altered DNA methylation in the intron region associated with CMT was comparatively investigated in both CMT and human breast cancer. Finally, we tried to show the putative function of differentially methylated regions (DMRs) in the intron region on gene expression using motif analysis with validation in additional samples.

## Results

### Genome-wide methylation was profiled in 11 pairs of CMT and adjacent normal tissues via MBD sequencing

**Fig. 1:**
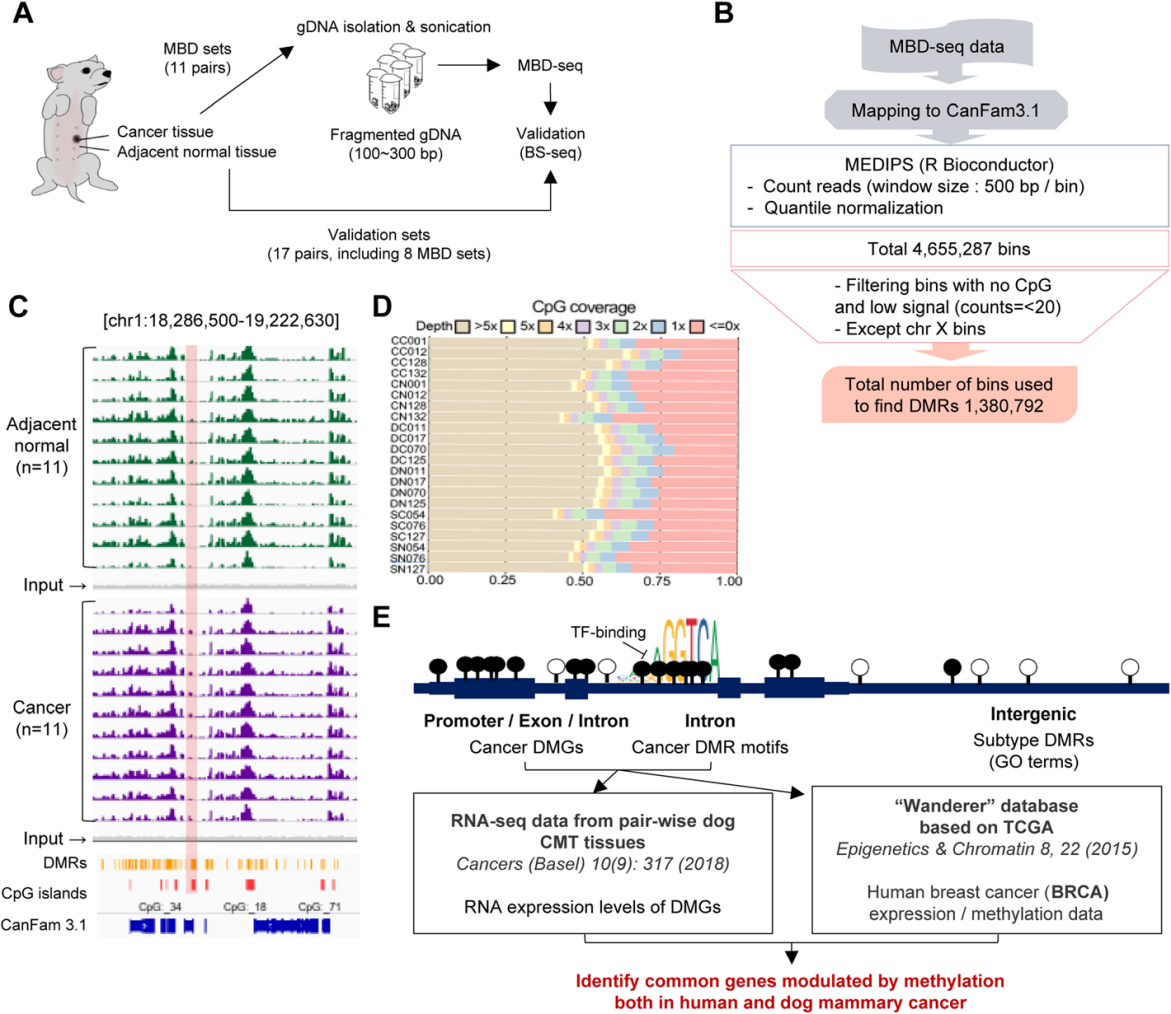
Schematic presentation of genome-wide methylation profiling in CMT using MBD sequencing. **a** Sample preparation for MBD-seq. **b** Sequencing data preprocessing with major parameters (window size 500 bp, filtration: bins without any CG, low signal: counts = <20, bins on Chr X). **c** Overall sequencing quality is visualized by IGV showing DMRs (yellow), CGI (red), and Gene information (blue). Methylation peaks are colored in 11 cancer (purple) and adjacent normal (green) samples. The region with high density of DMRs is highlighted by the red box. **d** High-quality signals (depth > 5X) cover more than 50% of the canine genome in 22 samples. **e** Analytical scheme of intergenic and genic regions or subtype-DMRs and CMT-DMRs. Additional data, CMT transcriptome and HBC expression and methylation, was investigated for further analysis

Eleven pairs of CMT and adjacent normal tissues consisting of three subtypes, simple, ductal, and complex carcinoma, were subjected to MBD sequencing (Fig. 1a, Table S1A). The statistic information, including the number of reads, Q20 and 30 scores for all the raw sequence data and enrichment scores, and the CG coverage for the processed sequence data generated in this study showed good quality (Table S2). From a total of 4,655,287 bins (500 bp in size), 1,380,792 high-quality bins were obtained by filtration of no CpG, low signals (counts = <20), and bins on the X chromosome (Fig. 1b). Even signal distribution across CMT and adjacent normal in the 11 samples was representatively depicted within the genomic region (Chr 1:18,286,500–19,222,630, ~ 100 Kb) by integrative genomic viewer (IGV) [26] with peak and annotation files. Differentially methylated regions (DMRs), shown in yellow, were distributed similarly on CpG islands and tended to be enriched in gene regions (Fig. 1c). The quality of MBD enrichment was checked according to the coverage of CpGs in the dog genome. Bins with high signal depth (> 5X) covered 45~55% of the dog genome, indicating that methylated DNA was successfully enriched by MBD not only from promoter regions but also from various regulatory regions, including both genic and intergenic regions (Fig. 1d). The methylation profiles were analyzed further by focusing on the DMRs in intergenic regions for the tissue origin of CMT subtypes and the DMRs in genic regions for CMT-enriched methylation. Gene ontology (GO) enrichment analysis and OncoScore [27] were employed to elucidate the functional linkage between differential methylation and gene regulation. Additionally, the transcription factor (TF) binding motifs on the subtype-enriched DMRs were investigated. The CMT-enriched methylation signatures and putative regulation were furthermore comparatively investigated in HBC datasets to show how epigenetically similar these two diseases are. The analytical scheme was depicted in Fig. 1e.

### Linearized mixed model (LMM) successfully clustered DMRs between CMT and adjacent normal, and among subtypes

**Fig. 2:**
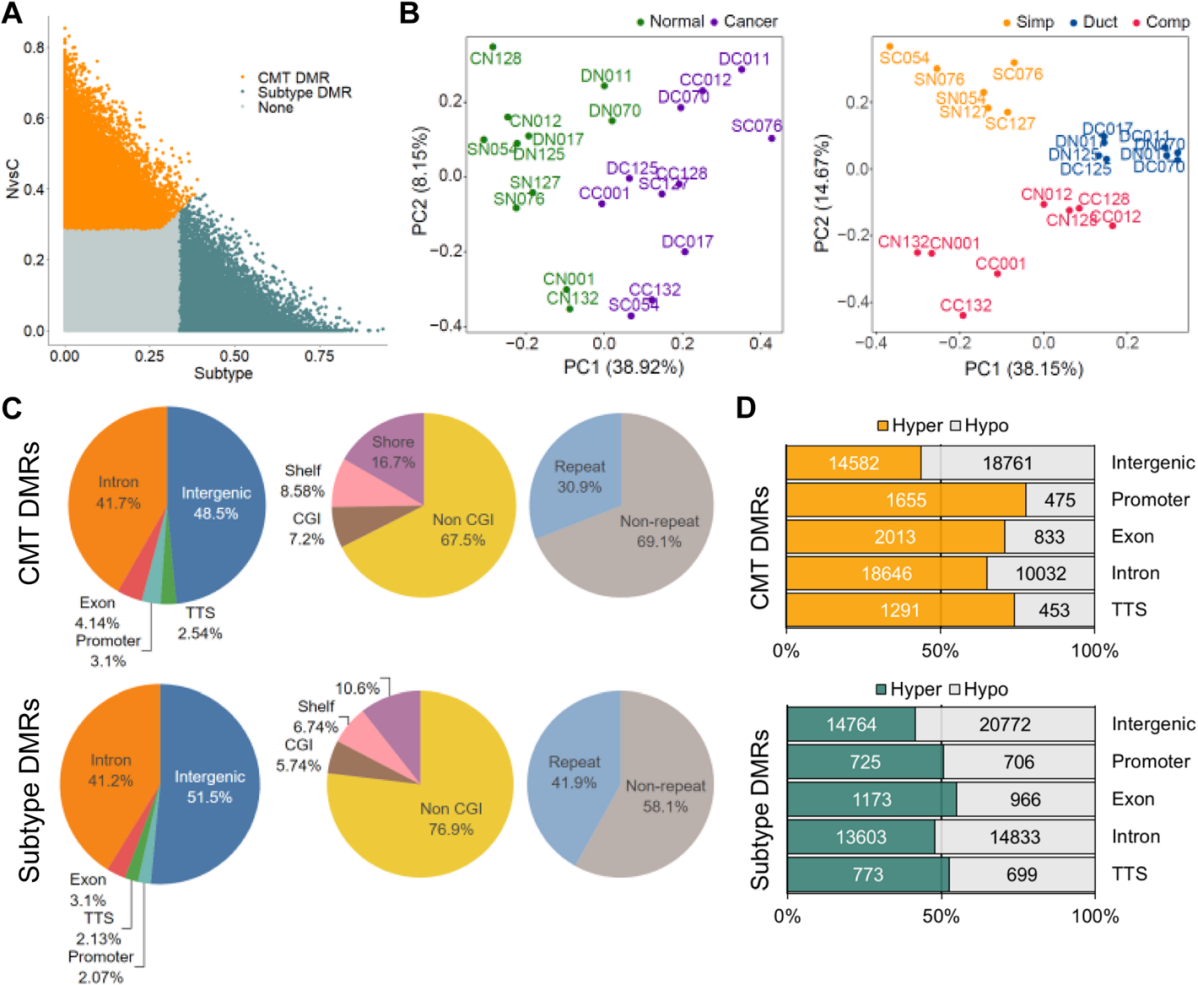
Identification of differentially methylated regions (DMRs) among the three CMT subtypes and between CMT and adjacent normal. **a** LMM separated CMT-DMRs (orange) and subtype-DMRs (blue green). Gray indicates none. **b** PCA analysis using CMT-DMRs and subtype-DMRs. CMT-DMRs successfully divides adjacent normal (green) and CMT (purple) and also subtype-DMRs into simple (orange), ductal (blue), and complex (red) types. **c** Genomic distribution of CMT-DMRs (up) and subtype-DMRs (down). Distribution between genic and intergenic regions (left), CGI and non-CGI (middle), and repeat and non-repeat (right). **d** Hyper- and hypomethylation profiles in CMT-DMRs and subtype-DMRs. Colored region (orange and blue green): hypermethylation, gray: hypomethylation

To determine differential methylated bins as variables that respond to CMT as well as each subtype, linearized mixed model (LMM) was employed and two different thresholds, top 5% and top 10% bins based on standard variation (SD) that corresponds to *p* value < 0.01 and *p* value < 0.05, respectively, were used to obtain DMRs. A total of 137,755 bins (68,741 for CMT DMRs and 69,014 for subtype DMRs) were determined as strict DMRs (5%) of either CMT or across subtypes (Fig. 2a and Table S3, 4). Principal component analysis (PCA) using the DMRs successfully separated 22 specimens with multiple variances (CMT and adjacent normal and three different subtypes: simple, ductal, and complex) into corresponding groups (Fig. 2b). The sum of PC1 and PC2 in both CMT- and subtype-DMRs represented more than 50% of the total DMRs. Although no clear difference was found in the comparison of genic features consisting of CMT- and subtype-DMRs, the non-CGI (CpG island) region showed a clear difference between CMT (67.5%)- and subtype (76.9%)-DMRs that might occur in the alteration of repeat element regions (30.9% in CMT-DMR/41.9% in subtype-DMR). On the contrary, the proportion of CGI (7.2%) and shore (16.7%) regions encompassed in CMT-DMRs was higher than in subtype-DMRs (CGI (5.74%) and shore (10.6%)) (Fig. 2c). Interestingly, methylation profiles (hyper- and hypomethylation) showed a distinct difference between CMT- and subtype-DMRs, although, no significant difference was seen in genome-wide methylation distribution. Of note, methylation patterns were clearly biased in genic regions of CMT-DMRs. Approximately 66% of CMT-DMRs in the genetic regions were hypermethylated, while only 45% of DMRs in the intergenic region were hypermethylated. This bias was not seen in subtype-DMRs, which indicates that the bias is not due to the MBD sequencing (Fig. 2d). This biased genic hypermethylation in CMT fits the general features of higher methylation of genic region in cancer tissues and is similar to a previous report in human BC by Ball et al. [28].

### Gene ontology (GO) enrichment and pathway analysis using DMRs on both genic and intergenic regions—fittingly represented the functional relationship between DMRs and CMT as well as subtypes

**Fig. 3:**
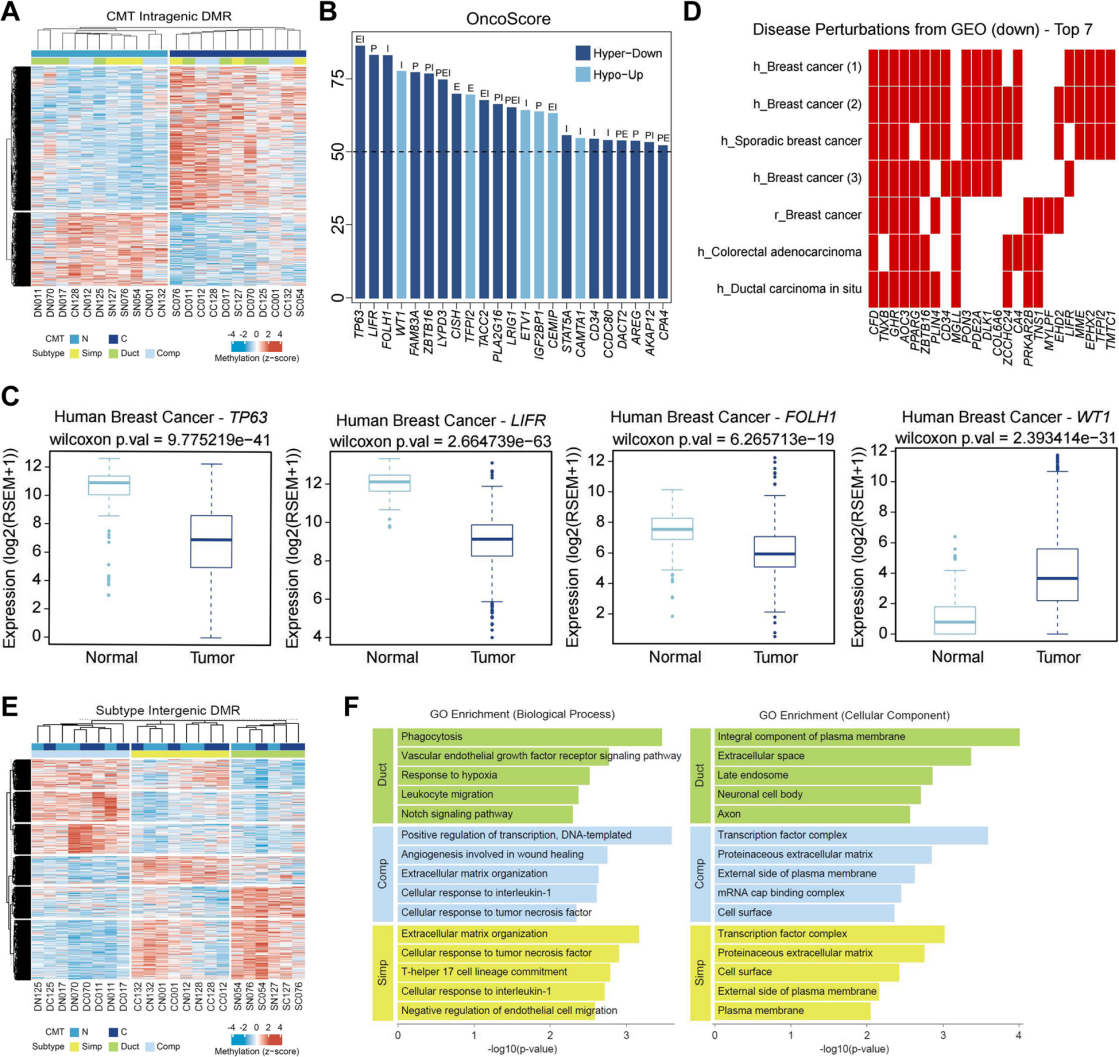
Functional association of DMGs. **a** Hierarchical clustering of CMT-DMGs separates 11 adjacent normal (light blue) and 11 CMT (dark blue) independent of subtypes (simple, yellow; ductal, green; and complex, blue). Methylation levels were z-scored and are indicated by blue (hypo) and red (hyper) scale. **b** OncoScore of 224 CMT-DMGs were measured and those with a score greater than 50 are depicted. Dark blue indicates hypermethylated DMGs and downregulated in RNA-seq data and light blue indicates hypomethylated DMGs and upregulated in RNA-seq data. **c** Box plot shows the expression level of the top 4 orthologous genes from the TCGA database ranked by OncoScore in normal (light blue) and human invasive breast cancer (dark blue). **d** CMT-DMGs were clustered into the library of Disease Perturbations from GEO (down). The top 7 terms are composed of breast cancer related terms. h, human; r, rat; m, mouse; (1) breast cancer C0006142 rat GSE1872; (2) breast cancer DOID-1612 human GSE26910; (3) sporadic breast cancer DOID-8029 human GSE3744; (4) colorectal adenocarcinoma DOID-0050861 human GSE24514; (5) tendonopathy 971 human GSE26051; (6) neurological pain disorder C0423704 rat GSE15041; and (7) ductal carcinoma in situ DOID-0060074 human GSE21422. See the Table S6 to show a list of terms and *p* values. **e** Hierarchical clustering of subtype-DMGs. **f** GO enrichment analysis in biological process (left) and cellular component (right). Duct, ductal; Comp, complex; and Simp, simple subtype. Length of bar represents − log 10 (*p* value)

Extraordinary hypermethylation throughout genic regions including promoter, exon, intron, and TTS in CMT was shown (Fig. 2d). On the other hand, differential methylation on intergenic regions where enhancers or silencers exist contributes to the tissue-type specificity [29]. We first performed hierarchical clustering and heatmap plotting using the genic regions of CMT-DMRs (Fig. 3a). Hypermethylation was more enriched in CMT than adjacent normal, parallel to Fig. 2d and what was previously known (Fig. 3a). Subsequently, OncoScore [27], functional annotations, and Gene ontology (GO) [30] enrichment analysis were performed with the list of CMT-DMGs (Fig. 3b, d and Table S5-S7) to investigate the functional linkage between DMGs and the molecular pathophysiology of CMT. As expected, many DMGs that were hypermethylated and downregulated in CMT including *TP63*, *LIFR*, *PLA2G16*, *LRIG1*, *STAT5A*, and *AKAP12* and have been known as tumor suppressors, were identified from high scoring (OncoScore > 50) CMT-DMRs (Fig. 3b). On the contrary, some oncogenes including *WT1*, *TFPI2*, and *ETV1* were also found from hypomethylated and upregulated DMGs. The methylation of 4 representative canine genes and their orthologous human genes, identified as three hypermethylated tumor suppressors (*TP63*, *LIFR*, and *FOLH1*) and one hypomethylated oncogene (*WT1*) in CMT, showed an anti-correlation with gene expression between normal and cancer in both dogs and humans (Fig. 3c and Additional file 1: Fig. S3). In addition, GO analysis with the disease perturbations from the GEO library revealed that CMT-DMGs were frequently enriched in the list of downregulated genes from various types of cancers including BC (breast cancer C0006142 rat GSE1872 sample 63 (*p* value = 1.4E− 16), breast cancer DOID-1612 human GSE26910 sample 602 (*p* value = 9.81E− 13), and sporadic breast cancer DOID-8029 human GSE3744 sample 979 (*p* value = 2.49E− 11)) (Fig. 3d). Furthermore, based on the methylation profiles in the intergenic regions of subtype-DMRs, the ductal subtype was distinctively separated from the simple subtype, while the complex subtype was located in between (Fig. 3e). This result may indicate that the cell type components are shared by the simple and complex subtypes of CMT but not by the ductal subtype. Hierarchical clustering was performed using the intergenic subtype-DMRs (Fig. 3e) and the nearest genes from the intergenic DMRs were found and processed with GO analysis. The list of genes near intergenic subtype-DMRs was presented in Table S8. The top 5 GO_biological process (BP) and GO_cellular component (CC) terms found in subtype-DMRs indicated that diverse processes were enriched in each subtype. Of note, simple and complex subtypes shared some biological processes, such as extracellular matrix organization (GO:0030198, *p* value = 6.79E− 04 (simple), *p* value = 2.32E− 03 (complex)) and cellular response to tumor necrosis factor (GO:0071356, *p* value = 1.25E− 03 (simple), *p* value = 4.56E− 03 (complex)), but all terms were unique in the ductal subtype, such as vascular endothelial growth factor receptor signaling pathway (GO:0048010, *p* value = 1.69E− 03). Similarly, in GO_CC, 4 out of 5 terms were also common in simple and complex subtypes, whereas all 5 terms in the ductal subtype were unique (Fig. 3f). This coincides with the hierarchical clustering in Fig. 3e. Substantial GO analysis using the nearest gene from intergenic CMT-DMRs as well as genic subtype-DMGs and pathway analysis using intergenic subtype-DMRs were performed and listed in Table S9-S11. In brief, no relevant terms to either cell types or cancer were retrieved (Table S9-S11).

### Aberration in intron methylation is associated with cancer

**Fig. 4:**
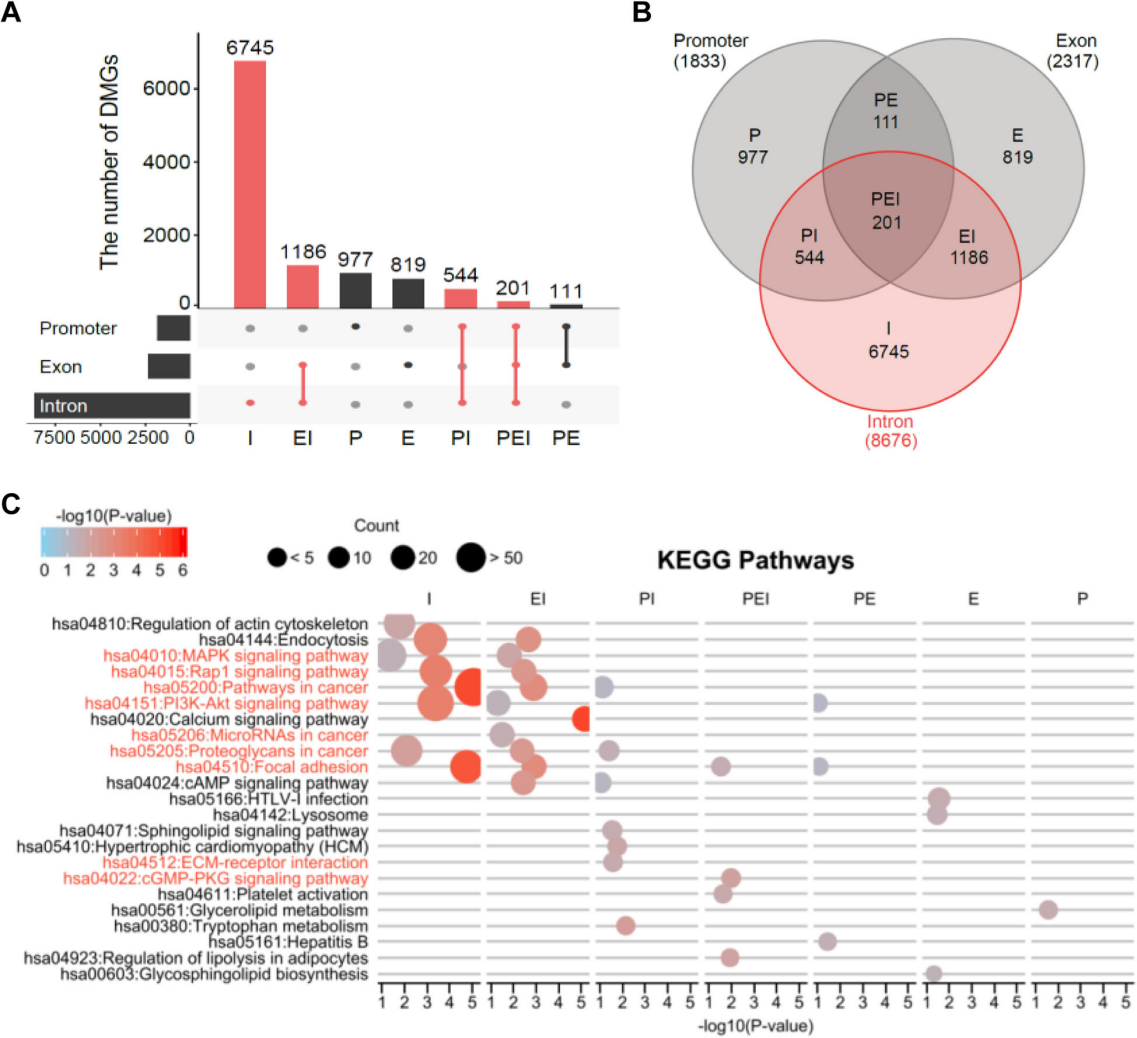
Intron DMRs may associate with cancer-related genes. **a** The DMGs are catagorized into 7 groups based on the combination of the DMR’s genic loci. I, intron only; EI, exon+intron; P, promoter only; E, exon only; PI, promoter+intron; PEI, promoter+exon+intron; and PE, promoter+exon. Red color indicates DMGs containing intron DMRs. **b** Venn diagram differentially presents intron DMRs (red) in 7 groups. **c** KEGG pathway analysis with intron DMRs shows cancer-related pathways are highly enriched in I and EI group. Both *x*-axis and gradient color indicates significance (− log10 (*p* value)), and the circle indicates the count of DMGs

A total of 10,583 CMT-DMGs were divided into 7 subgroups based on the distribution of DMRs (Fig. 4a). More than 60% of DMGs, consisting of 6745 genes, harbored DMRs only in the intron region, whereas 977 and 819 genes were identified with DMRs in only promoter and exon regions, respectively. A greater amount of intronic DMRs than either exonic or promoter DMRs could have been expected due to the large discrepancy in chromosomal coverage among the intron (26%), exon (1.5%), and promoter (< 1%) regions. Indeed, CMT-DMRs in the exon and promoter regions account for 22% and 17% of the total DMRs, respectively. This is higher than expected based on the coverage of the exon and promoter regions in the genomic sequence (less than 2%). This may mean that more CpG enrichment was done by MBD-seq in these areas (Fig. 4a).

The most interesting finding was that all terms associated with cancer in the Kyoto Encyclopedia of Genes and Genomes (KEGG) pathway analysis were enriched in DMRs that included intron DMRs such as intron only (I), exon+intron (EI), promoter+exon+intron (PEI), and promoter+intron (PI) (Fig. 4b, c). Not only the term of “pathways in cancer (hsa05200)” but also “microRNAs in cancer (hsa05206),” “proteoglycans in cancer (hsa05205),” “PI3K-Akt signaling pathway (hsa04151),” etc., which are associated with cancer and cancer pathophysiological characteristics, were highly enriched in intron only DMGs followed by EI and PI groups (Fig. 4c). However, KEGG terms such as “HTLV-1 infection (hsa05166),” “Neuroactive ligand-receptor interaction (hsa04080),” and “Lysosome (has04142)” that are extrinsic to cancer and CMT were enriched in DMGs that excluded intron DMGs such as the promoter only (P), exon only (E), and promoter+exon (PE) groups (Fig. 4c). Considering that intronic regions comprise a large portion of the genome, we counted the number of genes enriched in the “hsa05205: Pathways in cancer” term from a group of 530 genes, and the proportion for each group was calculated (data not shown). The percentage of cancer-related DMGs containing intron DMRs was 22.85% (I 5.34%, EI 7.80%, PI 6.70%, PEI 3.01%), which is higher than 17.27%, the percentage of cancer-related DMGs with promoter DMRs (P 3.51%, PI 6.70%, PE 4.05%, PEI 3.01%). Consequently, these results indicate that intron methylation may have important regulatory functions that are associated with CMT. It has been reported that intron CpG methylation might be associated with gene expression in human cancer. For instance, the methylation of the first intron of the *EGR2* gene, known as a tumor suppressor, affects the recruitment of proteins required for transcription [31], and anti-tumorigenic *PMP24* gene is silenced by the intronic single CpG methylation in prostate cancer cells [32].

### Altered CG methylation surrounding transcription factor binding motifs is an important epigenetic regulation in CMT

**Fig. 5:**
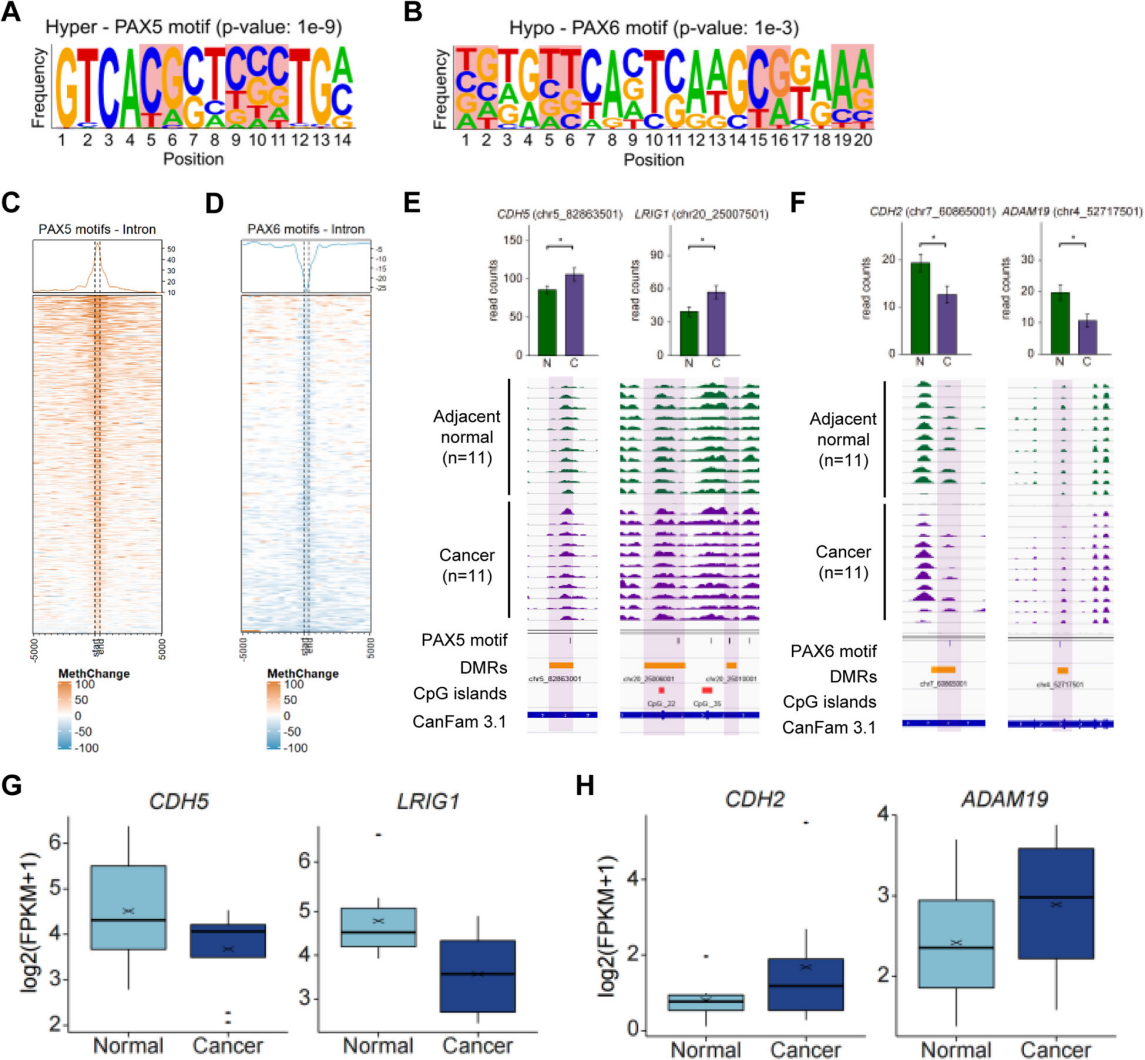
PAX motifs are enriched in hyper- and hypomethylated intron DMRs. Consensus motif sequence and sequence frequency of **a** PAX5 and **b** PAX6 motif. CGs on the motifs are highlighted with red. Accumulated heatmaps present 5 kb up- and downstream regions of **c** PAX5 and **d** PAX6 motifs. Hyper- (orange) and hypo- (blue) methylation. **e** and **f** Differential methylation peaks between 11 adjacent normal (green) and 11 cancer (purple) samples visualized with motif loci, DMRs, CGI, and gene structure annotations. *p* values for each DMR were generated by paired *t* test. The level of candidate gene expression (log2(FPKM+1) of **g***CDH5* and *LRIG1*, and **h***CDH2* and *ADAM19* in adjacent normal (light blue) and cancer (dark blue)

To investigate enriched CMT-responsible transcription factor (TF) binding motifs, intron DMRs were leniently extracted from the upper 10% of covariance in an LMM analysis (mean *p* value < 0.05, Figure S4A). The list of the top 10% of CMT-DMRs was also able to separately group cancer and adjacent normal (Figure S4B). According to the alteration of methylation, a total of 56,253 intron DMRs were obtained and subsequently divided into hyper- (36,401) and hypo- (19,852) methylated intron DMRs in CMT, then subjected to motif analysis using HOMER v4.11 [33]. Motif analysis revealed that 10 putative motifs, including PAX5, USF1, ZFX, and SREBF1, were enriched in hypermethylated intron DMRs, while 6 motifs, including CREB1, ELK1, PAX6, and ELK4 motifs, were enriched in hypomethylated intron DMRs. These motifs harbor CG nucleotides the methylation of which may influence protein binding activity [34]. We indeed focused on two PAX motifs, PAX5 and PAX6 that have been known as tumor suppressive and oncogenic, respectively [35–38]. Additionally, Kaplan-Meier plot [39, 40] showed breast cancer patients with lower PAX5 expression live shorter than those with higher, while the survival rate of patients with higher PAX6 expression decreased compared to those with lower expression (Fig. S5). It was expected that these two genes would have reverse effects in breast cancer. PAX5 and PAX6 motifs, respectively designated by 16 bp and 20 bp consensus nucleotide sequences (PAX5—GCAGCCAAGCGTGACC, PAX6—NGTGTTCAVTSAAGCGKAAA), were significantly enriched in each DMR group (PAX5 *p* value 1E− 9, PAX6 *p* value 1E− 3) (Fig. 5a, b and Table S12, S13). An enriched heatmap successfully visualized the enrichment of hyper- and hypomethylation signals in the 5 kb surrounding PAX5 and PAX6 motifs, respectively (Fig. 5c, d). We then investigated putative target genes that harbor hypermethylated PAX5 and PAX6 motifs in their intron regions (Table S14-S16). Hypermethylation in the intron DMRs of the PAX5 motifs of CMT, relative to that in adjacent normal, was visualized in the representative genes, *CDH5* and *LRIG1*, by IGV (Fig. 5e). On the other hand, hypomethylation related to PAX6 was found in the *CDH2* and *ADAM19* genes (Fig. 5f). All of these target genes, hyper- and hypomethylated in CMT, were reversely correlated to gene expression. RNA expression levels of the candidate genes were obtained from our previous transcriptome data [23] and an anti-correlation was shown by box plot (Fig. 5g, h). Unfortunately, in contrast to CMT-DMRs, no significant motifs were commonly enriched in subtype-DMRs (Table S17).

### Validation of intron DMRs and their anti-correlation to gene expression

**Fig. 6:**
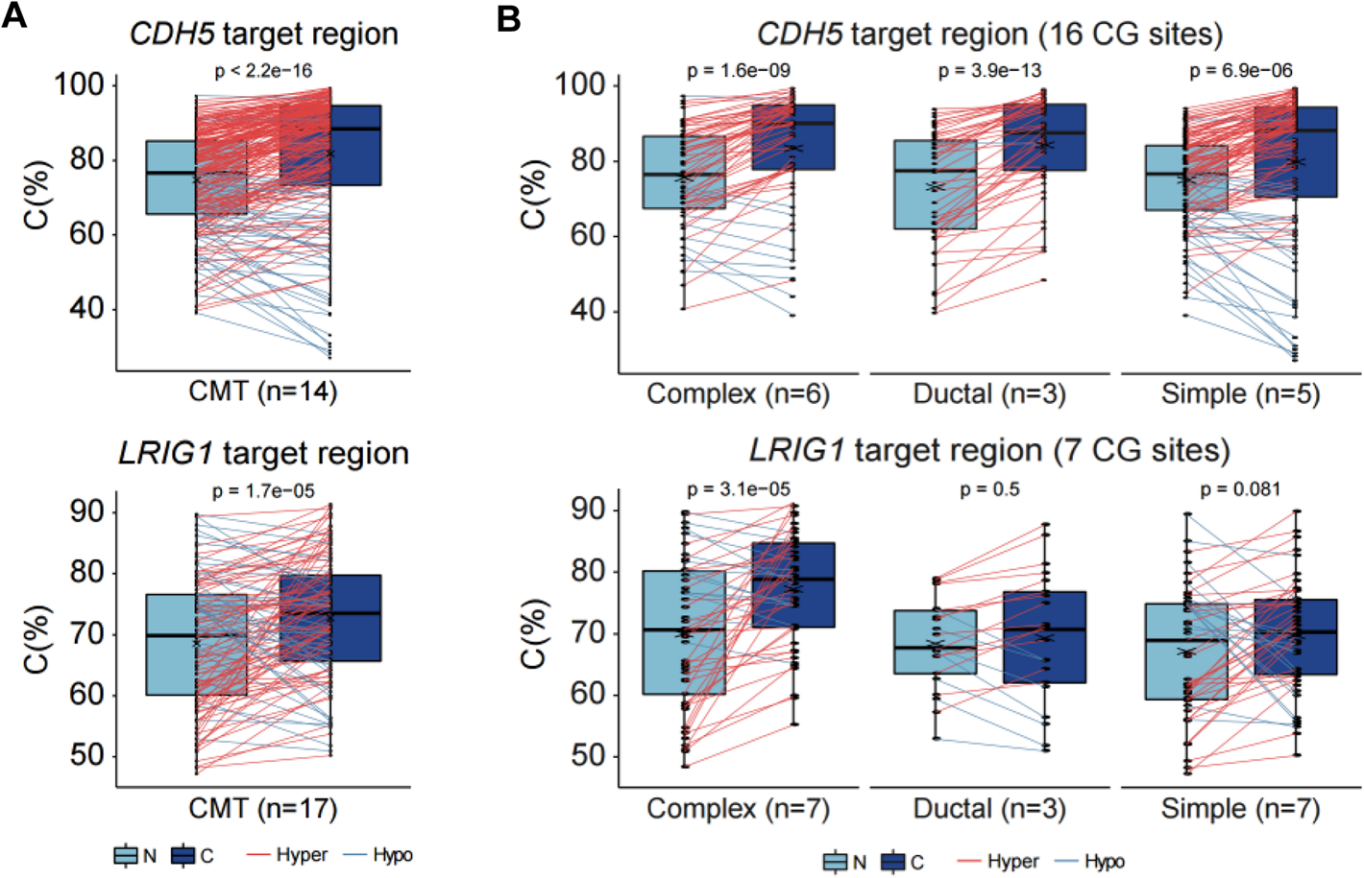
Validation of intron hypermethylation in the candidate genes, *CDH5* and *LRIG1*. **a** Comparison of overall methylation states in the surrounding regions of the intronic PAX5 motif in *CDH5* and *LRIG1* genes. Methylation was measured by the ratio of cytosine on each CG site. Red lines between CMT and adjacent normal indicate hypermethylation, while blue lines indicate hypomethylation. N, adjacent normal; C, CMT. Statistical *p* value was calculated by paired *t* test. **b** Differential methylation is depicted in three separated CMT subtypes

The methylome signature in CMT identified by MBD sequencing was validated in both the 8 pairs of specimens originally subjected to high-throughput sequencing and 9 additional validation sets. Bisulfite genomic DNA conversion followed by PCR was performed in the pairs of CMT and adjacent normal samples to obtain a fine map of intron methylation surrounding PAX5 motif regions of candidate genes (Table S18). Primers used in BS-conversion PCR and sequencing are listed in Table S19. Overall, a hypermethylated intron was confirmed in two candidate genes that included the PAX5 motif, *CDH5* and *LRIG1*, with box plots showing the DNA methylation profiles of the intron DMRs of genes (Fig. 6, Table S20). As for the *CDH5* and *LRIG1* genes, respectively, a total of 16 CGs and 7 CGs surrounding PAX5 motifs, were tested in 14 and 17 pairs of CMT and adjacent normal samples. Of the 16 CGs tested in the 1st intron region of *CDH5*, 12 showed significant hypermethylation (Fig. 6a, upper panel). Unexpectedly, the PAX5 motif was located on the 14th and 15th CGs where no significant difference was found (Fig. S6A). Pairwise comparison of each CG’s methylation between CMT and adjacent normal showed significant hypermethylation. In the intron-DMR tested region of *LRIG1*, all CG loci tended to show hypermethylation in CMT and one CG locus (1st CG, *p* value = 0.019, Fig. S6B), among them showed a significant difference (Fig. 6a, lower panel). In addition, differential intron methylation of *CDH5* was clear in all three CMT subtypes but showed the best result in the ductal subtype (*p* value = 3.9E− 13). The differences in *LRIG1* intron methylation were more distinct in the complex subtype (*p* value = 3.1E− 05) than in the other subtypes (Fig. 6b). These results suggest that hypermethylation of these two intron regions can be useful candidate epigenetic markers for CMT as well as subtypes.

### CMT-enriched differential intron methylation and its anti-correlation with gene expression was conserved in human breast cancer

**Fig. 7:**
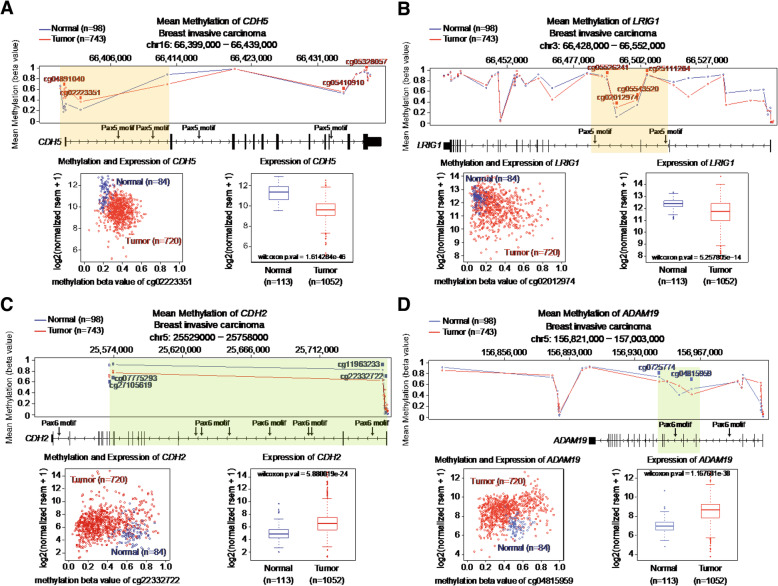
Conservation of intron DMRs and associating RNA expression in the candidate genes between HBC and CMT. Hypermethylated candidate genes, **a***CDH5* and **b***LRIG1*. Hypomethylated candidate genes, **c***CDH2* and **d***ADAM19*. Human gene structures are line-drawn with intron PAX5 and PAX6 motifs (arrows). Wanderer database provided CG methylation levels in normal (blue line) and cancer (red line). CGs surrounding PAX motifs are labeled in red (hypermethylation) or in blue (hypomethylation). Scatter plot presents anti-correlation between methylation level in selected CG and gene expression; normal: blue, cancer: red. Box plot shows overall gene expression levels of normal (blue) and cancer (red) in TCGA database

To validate our CMT-enriched methylome signature findings to human breast cancer (HBC), we investigated the consistency of the aberrations of candidate gene methylation and RNA expression between CMT and HBC. The methylation status and expression profiles of 4 representative candidate genes in HBC was surveyed using the Wanderer database (Fig. 7) [41]. We determined locally corresponding CG sites and introns of the human orthologous genes from the breast cancer methylome data. Methylation levels were regionally dynamic within a target gene and there were some CGs differentially methylated between normal and HBC populations (Fig. 7, top panels of mean methylation). The scatter plots for *CDH5* and *LRIG1* consisting of hypermethylated intron motifs depicted the trend of increased methylation and decreased gene expression in HBC when compared to normal and thus resulted in normal being represented by the blue dots located in the top-left and HBC being represented by the red dots located in the bottom-right (Fig. 7a, b). On the contrary, *CDH2* and *ADAM19* showed the opposite pattern of methylation profiles and gene expression between normal and HBC (Fig. 7c, d). Methylation profiles and gene expression of two *CDH* genes (hypermethylation in *CDH5*, hypomethylation in *CDH2*) were well-conserved in normal and HBC populations. The 1st intron of *CDH5* harboring the hypermethylated PAX5 motif in CMT was also hypermethylated and downregulated in HBC (Fig. 7a). Moreover, the 2nd intron of *CDH2* which harbors a hypomethylated PAX6 motif in CMT was also hypomethylated and upregulated in HBC (Fig. 7b). Of note, *LRIG1* has somewhat different gene structures in human and dog, such as different number of exons (22 in human, 25 in dog), and thus the hypermethylated intron with the PAX5 motif that has anti-correlation with gene expression (Fig. 7c) was found in the 3rd and 5th introns in human and dog, respectively. Similarly, hypomethylated PAX6 motifs in *ADAM19* have an anti-correlation with the gene expression even though the hypomethylated intronic PAX6 motifs are located on different introns in dog and human (13th intron in dog and 5th intron in human) (Fig. 7d).

As a whole, our date revealed that the orthologous intron regions of PAX5 and PAX6 binding motifs between human and dog have similar CG methylation alterations in breast cancers. These results thus suggest that the molecular similarity between CMT and HBC exists not only at the genomic and transcriptomic levels but also the epigenomic level.

## Discussion

The study of CMT has gained increasing importance not only for animal welfare but also for better understanding of HBC. Over the past decade, comparative studies of CMT and HBC have been conducted at the genome and transcriptome levels using high-throughput sequencing data and have presented similarities and discrepancies existing between CMT and HBC [23, 25]. However, a comprehensive analysis of the genome-wide methylome in CMT and its comparison with the HBC methylome had not been studied yet.

We employed a linearized mixed model to classify DMRs with multiple variances and successfully determined CMT- and subtype-DMRs. Our methylome data showed that DMRs were biased towards hypermethylation on the genic regions represented by promoter, exon, intron, and TTS in CMT. This is consistent with the previous knowledge that the general cancer methylation pattern is represented by intergenic hypomethylation and gene body hypermethylation [28]. In addition, each DMR (CMT- and subtype-) as a methylation signature could separate either normal from CMT or among the three subtypes in principal component analysis. The OncoScore and the GO enrichment analysis results demonstrated that our CMT- and subtype-DMRs are functionally linked to CMT and subtypes.

Of further note in the present study was that most of the enriched cancer-associated pathways were from DMRs that included intron regions. Recently, the regulatory role of the intron region has been proposed in certain gene expressions, particularly the first intron closely located to the promoter [31, 42, 43]. Some studies proposed enhancer sequences in introns and showed the transcription factor (TF) binding to the sequences [44]. Although, some studies also proposed alternative splicing in RNA causing intron retention as putative roles of intron DNA methylation, this needs to be further elucidated [42, 45, 46]. Furthermore, the role of TFs and DNA methylation in intron regions also needs to be elucidated because, although DNA methylation is generally associated with transcriptional silencing, the effect of methylation on binding affinity for most TFs is still unknown [47, 48]. Yet, Yin et al. measured the TF binding affinity to the methylated motif in about half of human TFs using modified high-throughput sequencing and suggested that the affinity of individual TFs can either be increased or decreased on methylation, depending on the different positions within the binding site [34]. In this study, we identified PAX5 and PAX6 motifs, known to be tumor suppressive and oncogenic TFs that are enriched in hyper- and hypomethylated intron DMRs of CMT, respectively. Nine members are known in the paired box (PAX) gene family and some members [49] particularly PAX5 and PAX6 are known to have similar binding sites based on their crystal structure [50]. However, recent studies provided enough evidence that PAX5 and PAX6 work independently [36–38]. For instance, they are clustered in different groups (PAX5 in group 2, PAX6 in group 4) [51] and bind to different genomic loci in ChIP-seq analysis [52]. It is also known that only PAX genes from the same group are capable of complementing the loss of function in others [51]. We also identified a list of motifs, such as NR2F1, RORA, HNF4G, NR3C, MYB, and RUNX that were enriched in intron DMRs but of which the motifs lacked a CG nucleotide inside their recognition sites. The substantial putative target genes reversely regulated by intron methylation around motifs were listed in Table S16. These are also meaningful to study further since these motifs without a CG sequence in their recognition site can still be influenced by the surrounding CG methylation levels [34].

There exists some limitation in directly comparing our CMT methylation profile to the HBC methylome database since the methylation profiling for HBC provided by TCGA was generated from an Infinium Human Methylation450 BeadChip array (Illumina, USA), not MBD sequencing. Nonetheless, the result showing the correlation between methylation in the intron region and gene expression may support the importance of intron methylation, at least in regard to these candidate genes, *CDH5* and *LRIG1* with PAX5 motifs and *CDH2* and *ADAM19* with PAX6 motif in both CMT and HBC (Fig. 7).

## Conclusion

In the present study, we first comprehensively profiled CMT methylation and inspected its correlation with the HBC methylome. We successfully separated CMT-DMRs and subtype-DMRs, and showed their biological relevance by GO and pathway enrichment analysis. We also suggested that changes in intron methylation play an important role in CMT by altering TF binding affinity. The importance of the intron methylation was further confirmed in the HBC data by anti-correlation of selected gene expression with intronic hypermethylated PAX5 and hypomethylated PAX6 motifs. This study allows us to better understand both HBC and CMT at the epigenomic level, yielding new insight into cross-species mechanisms of cancer initiation and progression by DNA methylation alteration and also into the development of cancer biomarkers.

## Materials and methods

### Tissue samples

Based on the methods reviewed and approved by the Seoul National University Institutional Review Board/Institutional Animal Care and Use Committee (IACUC SNU-170602-1), a total of 11 dog patients with clinically diagnosed CMT were enrolled in the present study. Tumor and adjacent normal tissue samples of spontaneously occurred canine mammary gland cancer were obtained and freshly frozen. The information for CMT dogs is provided in Table S1.

### Genomic DNA isolation and MBD sequencing

Genomic DNA was extracted from 11 pairs of CMT and adjacent normal tissues and sheared into 100–300 bp lengths using Bioruptor® Pico (Diagenode, Belgium). Methylated DNA fragments were captured by MBD-beads using the MethylMiner™ Methylated DNA Enrichment Kit (Cat# ME10025) from Invitrogen (CA, USA) according to the manufacturer’s protocol (Invitrogen, Carlsbad, CA). To obtain more highly methylated DNA, MBD-captured DNA was eluted step-wise with different NaCl concentrations (200, 300, 400, 600, and 800 mM) and ethanol precipitated. After that, we confirmed that methylated DNA was highly enriched in the 600 and 800 mM fractions using real-time PCR. We pooled the 600 and 800 mM fractions and then conducted paired-end sequencing (read length, 101 bp) on the Illumina Hiseq 4000 next-generation sequencing platform (Illumina, CA, USA) after library construction using the TruSeq Nano DNA Sample Preparation Guide (Part # 15041110 Rev. D) as the manufacturer’s guide.

### MBD-sequencing data processing

Both per base sequence quality and per sequence quality scores were checked with FastQC v0.11.8 (https://www.bioinformatics.babraham.ac.uk/projects/fastqc/) [53] and sequencing reads with low quality were trimmed using Trim Galore v0.5.0 [54]. Processed reads were mapped to the dog reference genome (CanFam3.1) with Bowtie2 v2.3.4.3 [55] and complete BAM files were obtained after converting SAM to BAM and removing duplicated reads in Linux OS. Using MEDIPS v.1.38.0 (R Bioconductor) [56], MBD reads were calculated in every bin, dividing the whole genome into user-defined window sizes (500 bp, total 4,655,287 bins). Each read per bin was quantile normalized to reduce experimental difference, followed by an estimation of genomic CpG coverage by sequencing depth (Fig. 1d), sequencing reproducibility (Fig. S1A), and enriched methylated fragments according to the number of CpGs in bins (Fig. S1B). Read counts across the total bins showed high correlation between each sample (Fig. S1C, S2A). The entire process is summarized in Fig. 1b.

### DMR identification using LMM (linear mixed model)

Bins located in chromosome X were excepted for downstream analysis because some CMT patients were spayed females, which could affect the methylation difference on sex chromosome. Low-signal bins with ~ < 20 counts throughout all samples and also bins with no CG dinucleotides had been removed to obtain only valuable signal peaks. Finally, a total of 1,380,792 bins were used for DMR identification. Covariance between “CMT vs. adjacent normal” and “between subtypes” respectively, were calculated for the entirety of the bins using R package “lme4” and we chose the upper 5% of the bins in each comparison group (between “CMT vs. adjacent normal” and “between subtypes”) following prioritizing variance by descending order from 0 to 1. After this, we defined bins whose priority between CMT vs. adjacent normal was higher than that between subtypes as “CMT-DMRs.” Inversely, if the priority between subtypes was higher than that between CMT vs. adjacent normal, we called those bins “Subtype-DMRs.” This LMM analysis and further analyses were performed using our own R script. *p* values and fold changes for DMRs were obtained using “MEDIPS.meth” function based on the “edge.R” calculation method.

### RNA expression

For 10 pairs of CMT dog tissues that we performed MBD-seq on in this study, RNA sequencing was also performed in a previous study and the data was obtained from PRJNA527698 (SRA accession number: SRR8741587-SRR8741602) [23]. Data processing was conducted as mentioned above (`Material and Methods - MBD-sequencing data processing`). Using “CuffLinks,” a tool to quantitate RNA expression data and statistically identify differential expression between groups, we estimated expression levels for 32,218 genes and identified DEGs based on *p* value (*p* < 0.05).

### OncoScore

OncoScore is a tool that scores genes according to their association with cancer, based on text-mining technology using the available scientific literature in PubMed. OncoScore for DMGs with anti-correlated expression was obtained through the R package “OncoScore” (https://github.com/danro9685/OncoScore) [27].

### Functional annotation

To investigate the disease enrichment analysis in Fig. 3d, we used the interactive web-based enrichment analysis tool, “Enrichr” (http://amp.pharm.mssm.edu/Enrichr/) [30, 57]. Among 35 gene set libraries in Enrichr, a category of the Disease Perturbations from GEO (Gene Expression Omnibus) down was chosen to find the disease terms. We investigated the functional annotation of 7 DMG groups (Fig. 4a) and searched for subtype-associated GO terms using “DAVID,” a web-based software for functional annotation analysis (https://david.ncifcrf.gov/summary.jsp) [58]. Since the database of gene ontology in dog is not well established, we converted the dog Ensembl Gene IDs to human IDs using the table of human-dog gene orthologues provided by Ensembl BioMart (www.ensembl.org/biomart/martview) [59]. The functional mechanism studies for dog genes are poorly conducted. KEGG terms for CMT DMGs with *p* values < 0.05 were considered relevant. Only the top 5 GO terms for each subtype are shown in Fig. 2f.

### Motif analysis

Highly enriched known motifs in hypermethylated and hypomethylated intron DMR sequences were respectively identified using the “HOMER – findMotifsGenome.pl” command. The CpG normalization option was used since genome-wide methylation changes in CMT usually occur in CpG-rich regions. The *p* value for each motif was estimated by comparing the percentage of target sequence with motifs with the percentage of background sequence with motifs. We considered motifs relevant when the *p* value was < 0.01. After that, we found loci where the PAX5 and PAX6 motifs exist across the dog reference genome “CanFam3” (or “hg19” for human) using a motif scanning tool, “FIMO” (matched *p* value < 0.01) (http://meme-suite.org/doc/fimo.html).

### Targeted BS-conversion sequencing

A total 17 pairs of CMT and adjacent normal tissue were used for validation, including the same 8 sets used in MBD sequencing (Table S1B). Bisulfite conversion was done on 500 ng of genomic DNA using the EZ DNA Methylation-Lightning Kit (Zymo Research, USA). Primers were designed using MethPrimer (http://www.urogene.org/methprimer/index1.html) [60] and are listed in Table S19. After PCR, amplicons were purified from the agarose gels using the QIAquick Gel Extraction Kit (Qiagen, Germany) and directly sequenced at Macrogen Co. Ltd. (Macrogen Co. Ltd., Seoul, Korea).

### Human TCGA (BRCA) expression and methylation data

RNA sequencing and Infinium Human Methylation 450 K BeadChip array were performed in various human cancer types, such as human invasive breast cancer patients, and in normal people. Wanderer (http://maplab.imppc.org/wanderer/) grants access to a large dataset and offers an interactive viewer to show expression and methylation levels for interesting genes in BRCA (data for other cancer types also provided) [41]. We could thus obtain the methylation beta value for the interesting CGs near PAX motif regions of target genes (*CDH5*, *LRIG1*, *CDH2*, and *ADMA19*) and their transcription level changes in BRCA patients (Wilcoxon’s test).

### Statistical analysis

To estimate the methylated CpG level between CMT and adjacent normal tissues, we calculated the ratio of C/(C+T) from the BS-sequencing data. For validating methylation changes between them in the target motif DMR regions, statistical significance was assessed on *p* values obtained by paired *t* test using R basic command.

## Supplementary information

**Additional file 1: **Supplementary Figures. Figure S1. Data Quality Check for MBD sequencing. A) Saturation analysis for MBD-seq data from each sample was done using the MEDIPS package. Shown is the saturation analysis result derived from 22 MBD-sequencing samples. B) Coverage pattern analysis illustrates the fraction of CpGs covered by the given reads according to read depth. C) Pearson’s correlation for all counted peaks between experimental samples. Figure S2. Overview of DNA methylation peaks across samples throughout the full genome. IGV shows MBD-peaks (dark brown for 11 adjacent normal samples, dark blue for 11 cancer samples and input (gray) across the dog genome (Chr 1-38 and X). Differentially methylated bins (yellow), CpG islands (red) and gene annotations of CanFam3.1 (blue) are also displayed. Most of the peaks are well-enriched around CpG islands and genes and all experimental sets are performed with high similarity to each other. Figure S3. The expression level of the top 4 orthologous genes ranked by OncoScore in canine mammary tumor. Box plots show the expression level of four genes (*TP63, LIFR, FOLH1* and *WT1*) in adjacent normal (n=8) and paired CMT tissues (n=8). Expression values are presented as FPKM calculated from RNA-sequencing data. Statistical p-value was calculated by Wilcoxon’s test. Figure S4. Adjustment of thresholds to select distinguished CMT-DMRs for intronic motif analysis. A) P-values for each DMR was extracted using a serial cutoff manner (upper 1~20%), B) Dendrogram for 22 cancer and adjacent normal tissue samples separate cancer groups from normal when CMT-DMRs are identified at the 10% cutoff in linear mixed model (LMM). Figure S5. Kaplan-Meier plots showed PAX5 and PAX6 expression’s reverse effect on the survival rate of breast cancer patients. Survival rates depend on A) PAX5 and B) PAX6 expression. Web-based KM-plotter (https://kmplot.com/analysis/index.php?p=service) was used for drawing KM plots. Figure S6. Validation of individual CG methylation around PAX5 motif regions in *CDH5* and *LRIG1* genes. Paired t-test for individual CG in A) *CDH5* and B) *LRIG1* intronic PAX5 motif region. Percentage of methylated cytosine (C (%)) is represented by (C/C+T) * 100. Red lines between CMT and adjacent normal indicate hypermethylation, while blue lines indicate hypomethylation (N: adjacent normal, C: CMT). Statistical p-value was calculated by paired t-test.

**Additional file 2: **Supplementary Tables. Table S1. Information for CMT tissue samples. Table S2. Quality check for MBD-sequencing. Table S3. List of CMT-DMRs (68,741) and their genomic features. Table S4. List of Subtype-DMRs (68,741) and their genomic features. Table S5. Differentially methylated genes anti-correlated with expression. Table S6. Functional annotations for CMT-DMGs in 'Disease Perturbations from GEO down'. Table S7 GO terms in CMT-DMGs. Table S8. List of nearest genes from hypomethylated intergenic regions in each subtype. Table S9. GO terms in Subtype DMGs. Table S10. GO terms in the nearest genes from intergenic CMT-DMRs. Table S11. KEGG pathways in intergenic Subtype-DMRs. Table S12. Motifs enriched in hypermethylated intron bins (169, p-val < 0.01). Table S13. Motifs enriched in hypomethylated intron bins (83, p-val < 0.01). Table S14. Genes containing Pax5 motifs in their hypermethylated intronic regions. Table S15. Genes containing Pax6 motifs in their hypomethylated intronic regions. Table S16. Putative target genes with differentially methylated intron motifs. Table S17. Motif enrichment in each Subtype-DMR. Table S18. Validation target genes with differentially methylated PAX motifs. Table S19. Primers designed for BS-conversion PCR. Table S20. Validation of single CpG methylation by BS-seq in *CDH5* and *LRIG1* genes

## Data Availability

All MBD-seq data generated in this study have been deposited with links to BioProject accession number PRJNA601533 in the NCBI BioProject database (https://www.ncbi.nlm.nih.gov/bioproject/).

## Abbreviations

ADAM: A Disintegrin and Metalloproteinase
BP: Biological process
BRCA: Breast invasive carcinoma
CC: Cellular component
CDH: Cadherin
CGI: CpG island
CMT: Canine mammary tumor
DMG: Differentially methylated genes
DMR: Differentially methylated regions
DNMT: DNA methyl transferases
EGF: Epidermal growth factor
GO: Gene ontology
HBC: Human breast cancer
KEGG: Kyoto Encyclopedia of Genes and Genomes
LMM: Linearized mixed model
LRIG: Leucine rich repeats and immunoglobulin like domains
MBD-seq: Methyl-CpG binding domain sequencing
PAX: Paired box
PCA: Principal component analysis
SD: Standard variation
TCGA: The Cancer Genome Atlas
TF: Transcription factor
