# Supplementary material for: Alternative methylation of intron motifs is associated with cancer-related gene expression in both canine mammary tumor and human breast cancer
Source: Clin Epigenetics. 2020 Jul 21;12:110. doi: 10.1186/s13148-020-00888-4 (PMC7374976; doi:10.1186/s13148-020-00888-4)
Supplement: Supplementary file 1 — Additional file 1: Supplementary Figures. Figure S1. Data Quality Check for MBD sequencing. A) Saturation analysis for MBD-seq data from each sample was done using the MEDIPS package. Shown is the saturation analysis result derived from 22 MBD-sequencing samples. B) Coverage pattern analysis illustrates the fraction of CpGs covered by the given reads according to read depth. C) Pearson’s correlation for all counted peaks between experimental samples. Figure S2. Overview of DNA methylation peaks across samples throughout the full genome. IGV shows MBD-peaks (dark brown for 11 adjacent normal samples, dark blue for 11 cancer samples and input (gray) across the dog genome (Chr 1-38 and X). Differentially methylated bins (yellow), CpG islands (red) and gene annotations of CanFam3.1 (blue) are also displayed. Most of the peaks are well-enriched around CpG islands and genes and all experimental sets are performed with high similarity to each other. Figure S3. The expression level of the top 4 orthologous genes ranked by OncoScore in canine mammary tumor. Box plots show the expression level of four genes (TP63, LIFR, FOLH1 and WT1) in adjacent normal (n=8) and paired CMT tissues (n=8). Expression values are presented as FPKM calculated from RNA-sequencing data. Statistical p-value was calculated by Wilcoxon’s test. Figure S4. Adjustment of thresholds to select distinguished CMT-DMRs for intronic motif analysis. A) P-values for each DMR was extracted using a serial cutoff manner (upper 1~20%), B) Dendrogram for 22 cancer and adjacent normal tissue samples separate cancer groups from normal when CMT-DMRs are identified at the 10% cutoff in linear mixed model (LMM). Figure S5. Kaplan-Meier plots showed PAX5 and PAX6 expression’s reverse effect on the survival rate of breast cancer patients. Survival rates depend on A) PAX5 and B) PAX6 expression. Web-based KM-plotter (https://kmplot.com/analysis/index.php?p=service) was used for drawing KM plots. Figure S6. Validation of indi [file 13148_2020_888_MOESM1_ESM.pdf]

A

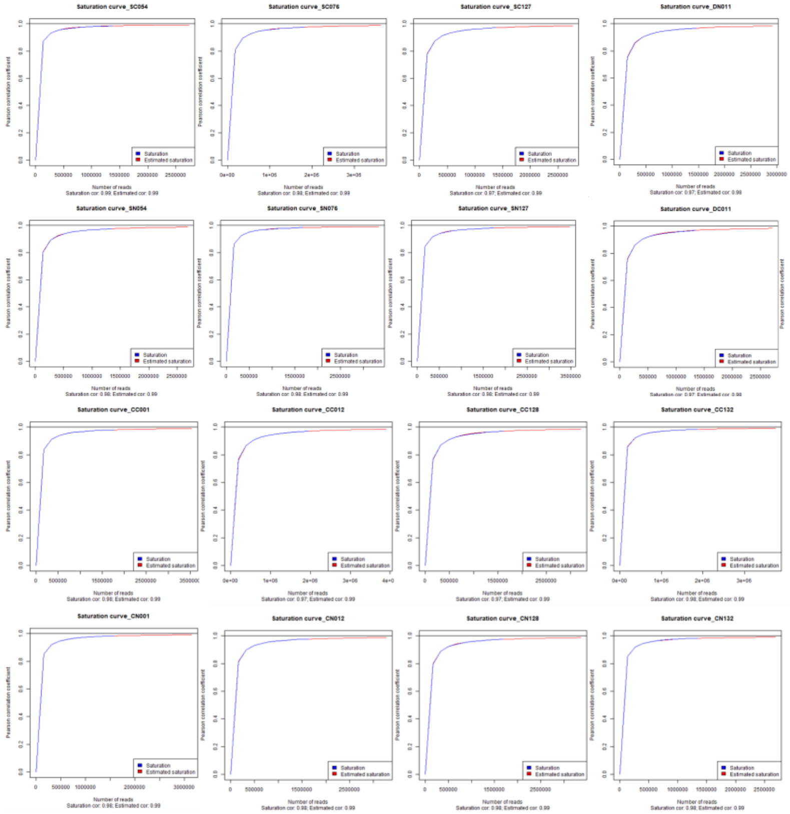
Saturation curve_SC054
Saturation curve_SC076
Saturation curve_SC127
Saturation curve_DN011
Saturation curve_DC017
Saturation curve_DC070
Saturation curve_DC125
Saturation curve_SN054
Saturation curve_SN076
Saturation curve_SN127
Saturation curve_DC011
Saturation curve_DN017
Saturation curve_DN070
Saturation curve_DN125
Saturation curve_CC001
Saturation curve_CC012
Saturation curve_CC128
Saturation curve_CC132
Saturation curve_CN001
Saturation curve_CN012
Saturation curve_CN128
Saturation curve_CN132

B

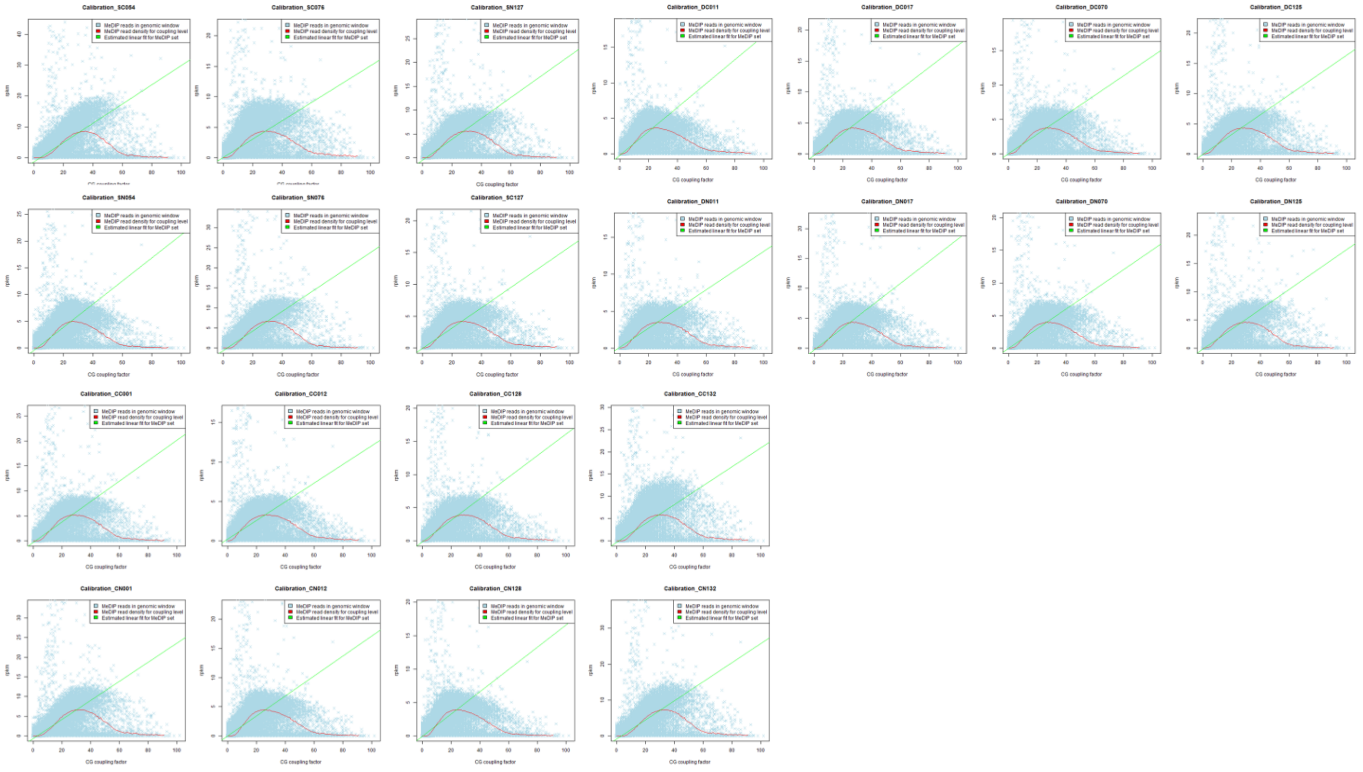
Calibration_SC054
Calibration_SC076
Calibration_SN127
Calibration_DC011
Calibration_DC017
Calibration_DC070
Calibration_DC125
Calibration_SN054
Calibration_SN076
Calibration_SC127
Calibration_DN011
Calibration_DN017
Calibration_DN070
Calibration_DN125
Calibration_CC001
Calibration_CC012
Calibration_CC128
Calibration_CC132
Calibration_CN001
Calibration_CN012
Calibration_CN128
Calibration_CN132

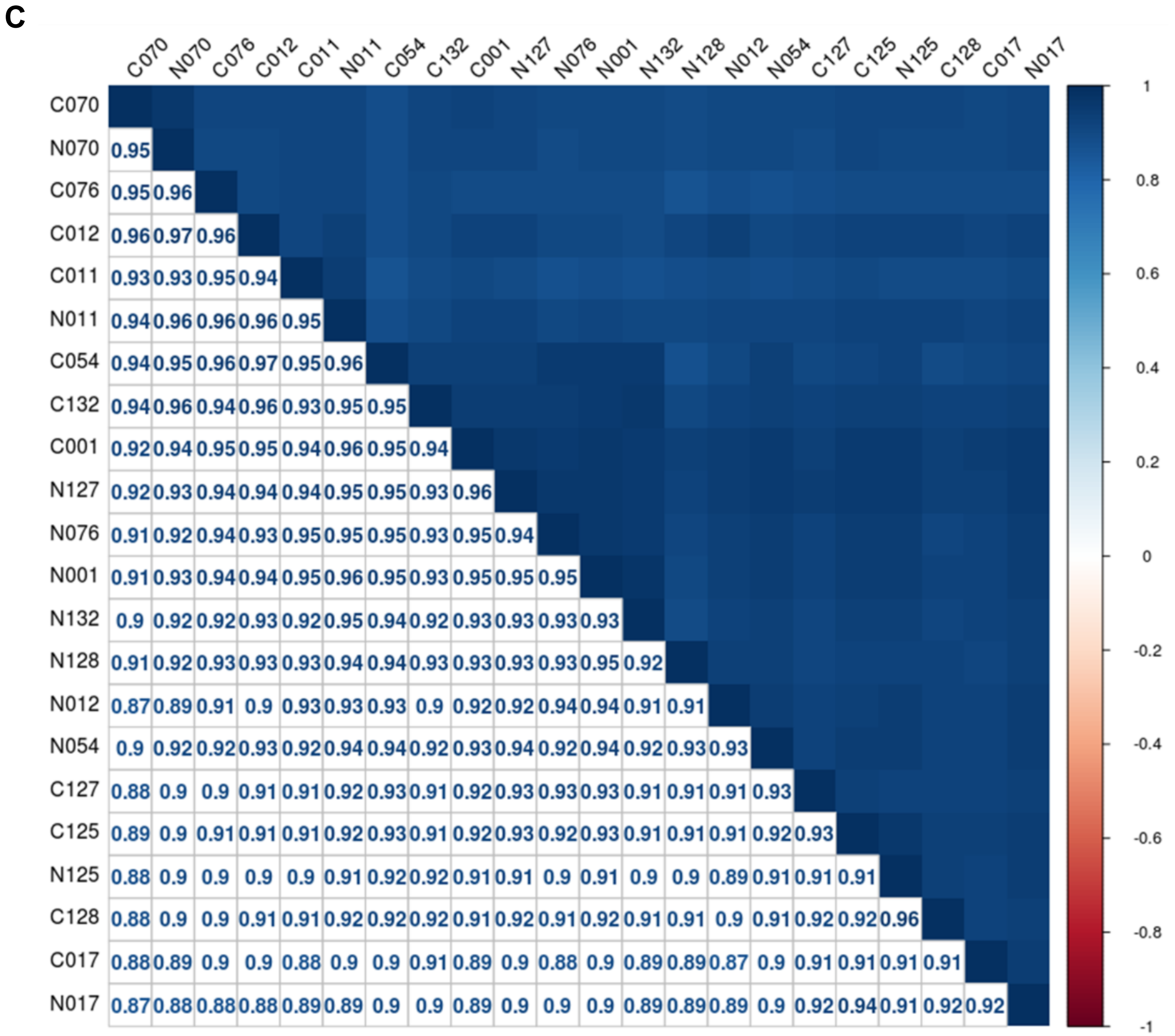

Supplementary Fig 1. Data Quality Check for MBD sequencing. A) Saturation analysis for MBD-seq data of each sample was done using the MEDIPS package. Shown is saturation analysis result derived from 22 MBD-sequencing samples. B) Coverage pattern analysis illustrates the fraction of CpG covered by the given reads according to read depth. C) Pearson's correlation for all counted peaks between experimental samples.

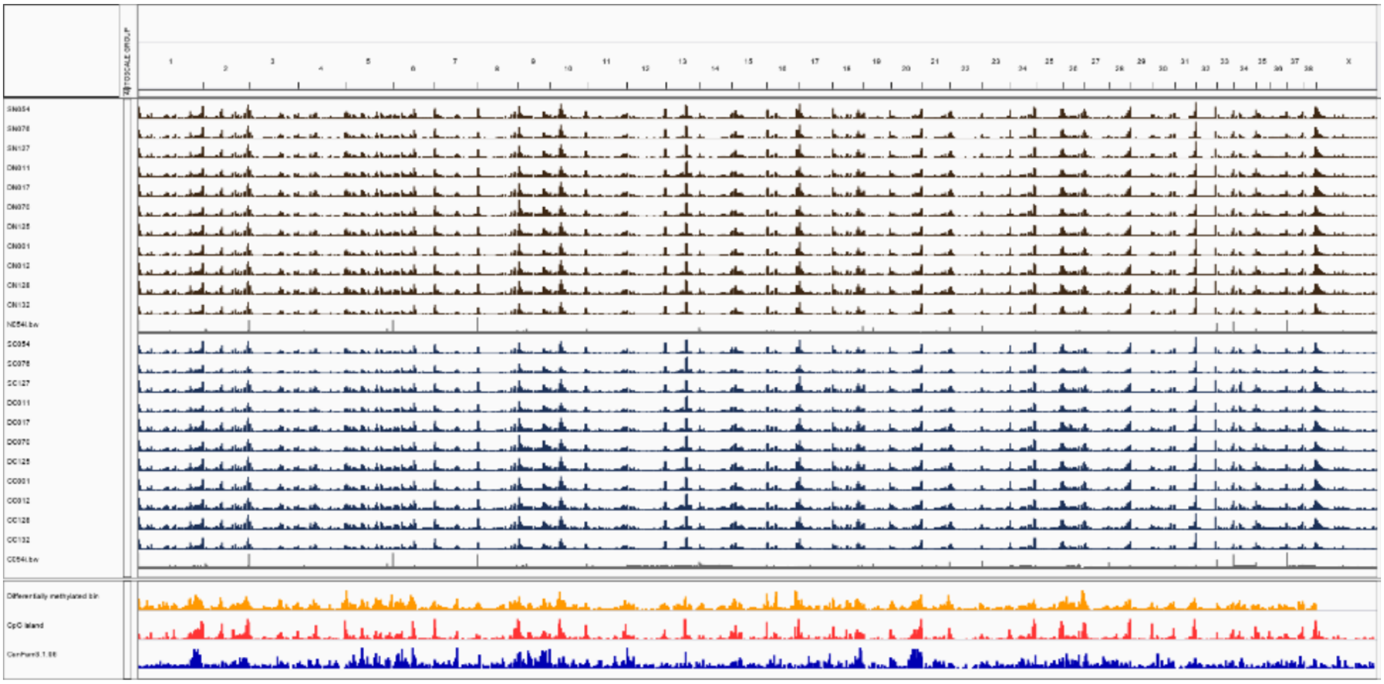

Supplementary Fig 2. Overview of DNA methylation peaks throughout the full genome. IGV showed MBD-peaks (dark brown for 11 adjacent normal samples, dark blue for 11 cancer samples and input (gray) across the dog genome (Chr 1-38 and X). Differentially methylated bins (yellow), CpG islands (red) and gene annotations of CanFam3.1 (blue) are also displayed. Most of peaks are well-enriched around CpG islands and genes and all experimental sets are performed with high similarity each other.

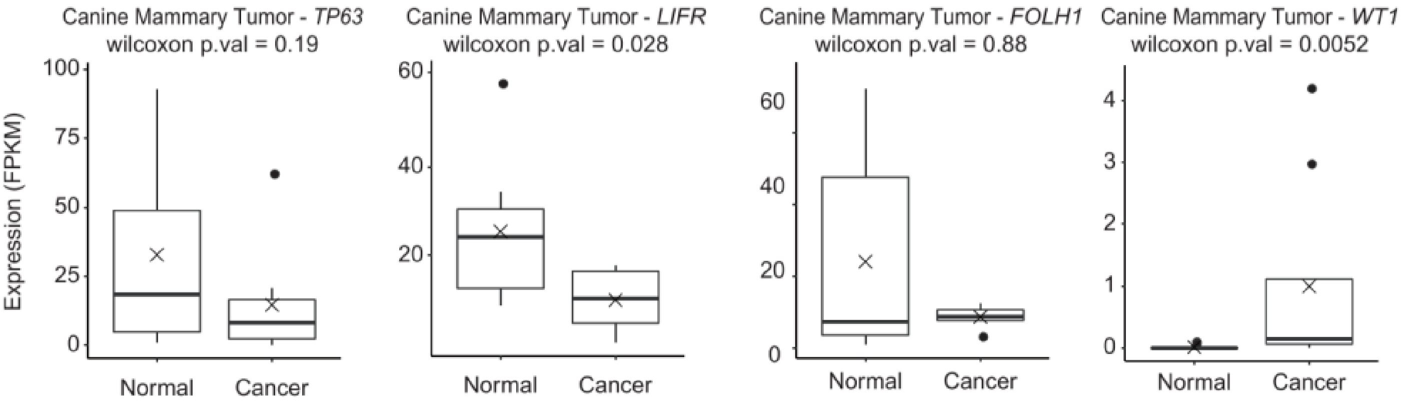

Supplementary Fig 3. The expression level of the top 4 orthologous genes ranked by OncoScore in canine mammary tumor. Box plots showed the expression level of four genes (*TP63, LIFR, FOLH1* and *WT1)* in adjacent normal (n=8) and paired CMT tissues (n=8). Expression values are presented by FPKM calculated from RNA-sequencing data. Statistical p-value was calculated by Wilcoxon's test.

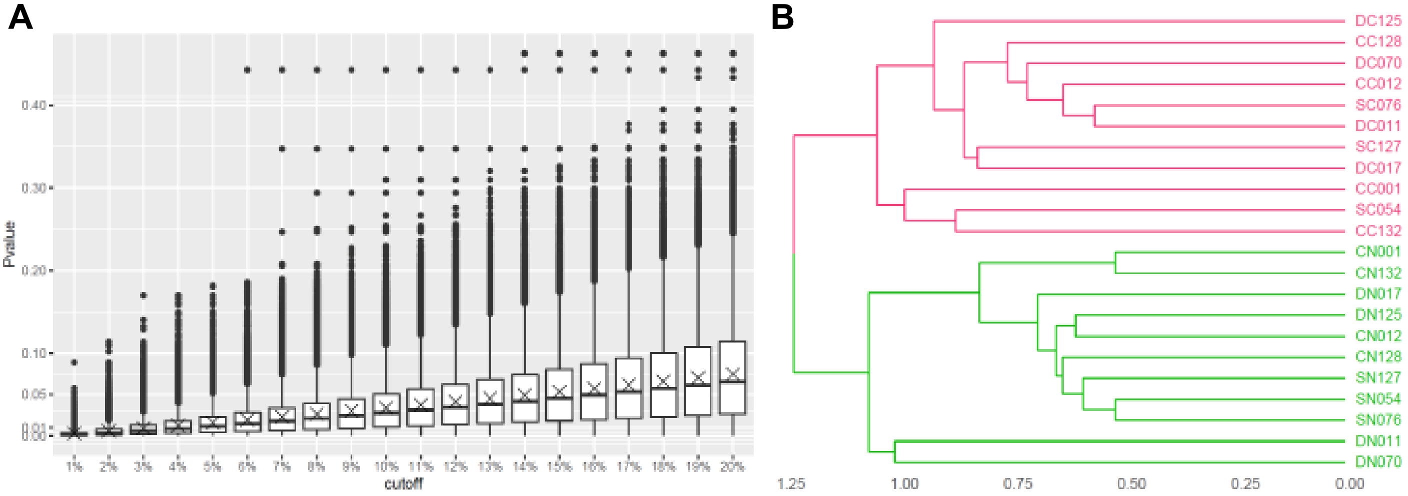

Supplementary Fig 4. Adjust thresholds to select distinguished CMT-DMRs for intronic motif analysis. A) P-values for each DMRs extracted using serial cutoff manner (upper 1~20%), B) Dendrogram for 22 cancer and adjacent normal tissue samples supervised cancer groups from normal when we identify CMT-DMRs at cutoff 10% in linear mixed model (LMM).

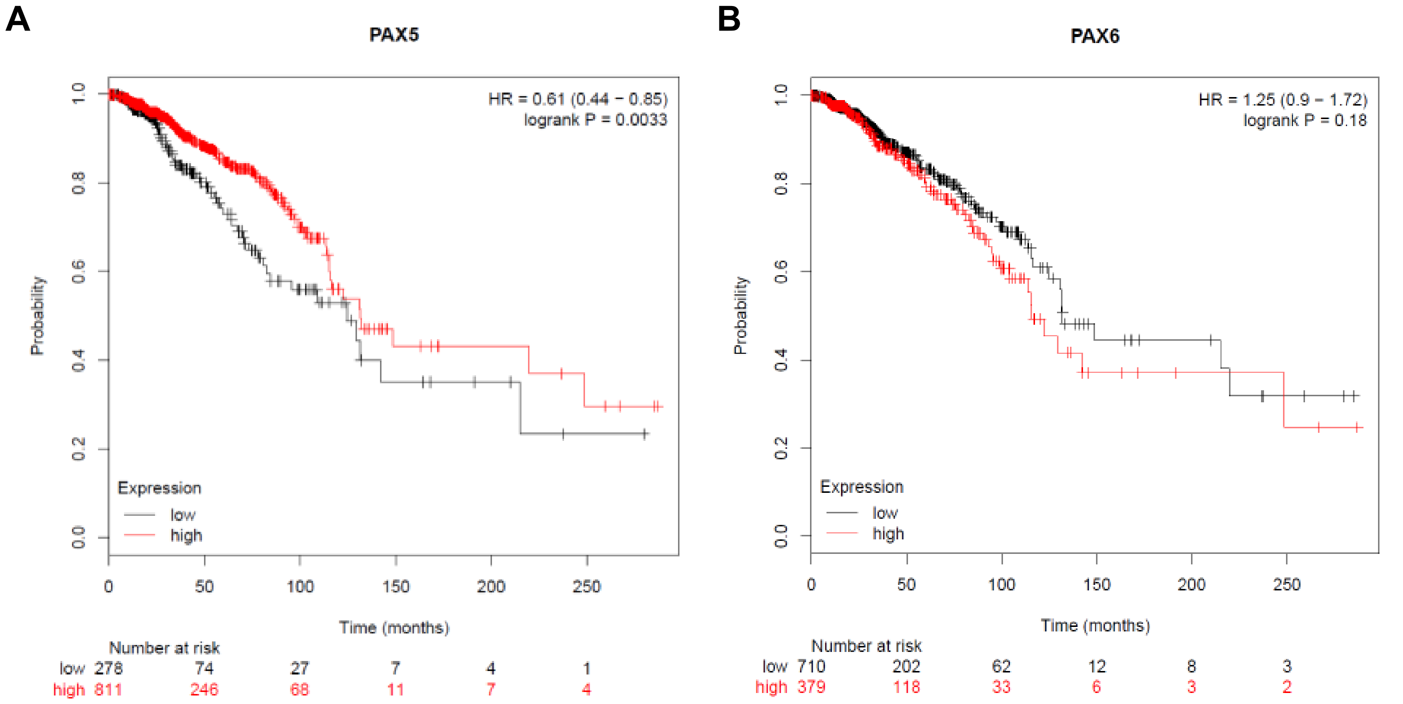

Supplementary Fig 5. Kaplan-Meier plots showed PAX5 and PAX6 expression reversely effect on the survival rate of breast cancer patients. Survival rates depends on A) *PAX5* and B) *PAX6* expression. Web-based KM-plotter (https://kmplot.com/analysis/index.php?p=service) was used for drawing above KM plots.

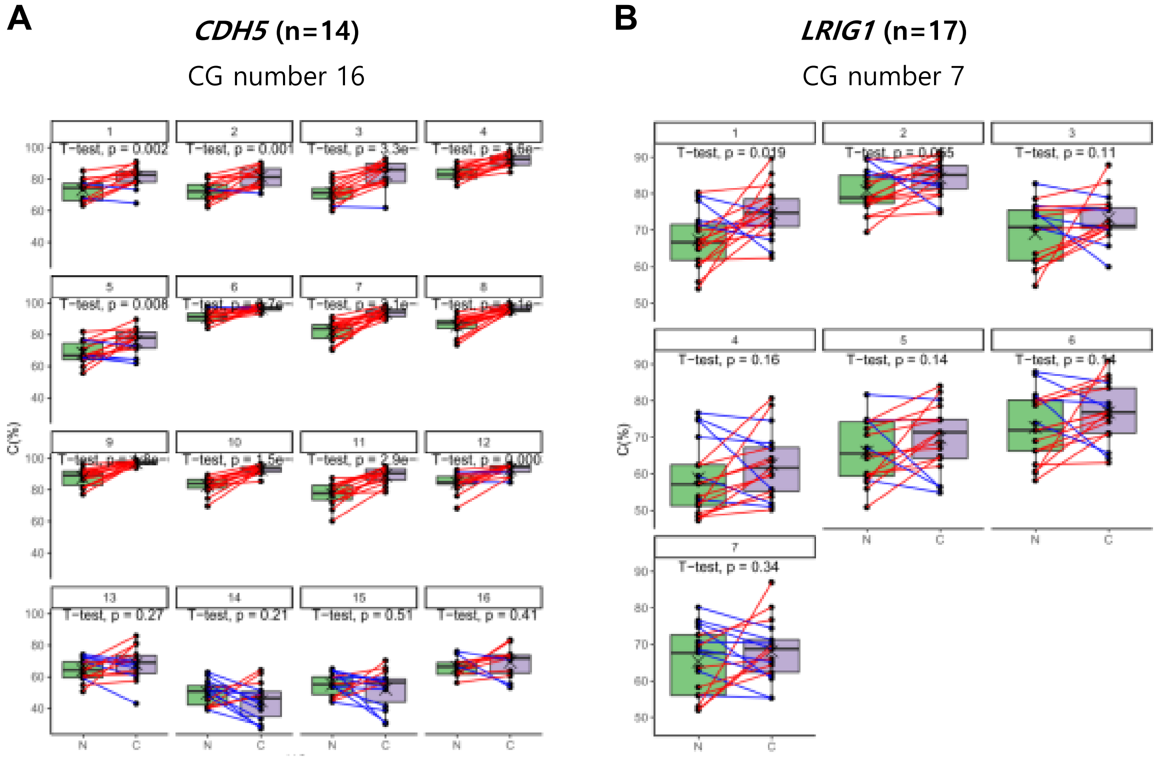

Supplementary Fig 6. Validation of individual CG methylation around PAX5 motif regions in *CDH5* and *LRIG1* genes. Paired t-test for individual CG in A) *CDH5* and B) *LRIG1* intronic PAX5 motif region. Percentage of methylated cytosine (C(%)) were represented by (C/C+T)*100. Red lines between CMT and adjacent normal indicate hypermethylation, while blue line indicate hypomethylation (N: adjacent normal, C: CMT). Statistical p-value was calculated by paired t-test.
